# Trends in leading causes of hospitalisation of adults with diabetes in England from 2003 to 2018: an epidemiological analysis of linked primary care records

**DOI:** 10.1016/S2213-8587(21)00288-6

**Published:** 2022-01

**Authors:** Jonathan Pearson-Stuttard, Yiling J Cheng, James Bennett, Eszter P Vamos, Bin Zhou, Jonathan Valabhji, Amanda J Cross, Majid Ezzati, Edward W Gregg

**Affiliations:** a Department of Epidemiology and Biostatistics, School of Public Health, Imperial College London, London, UK; b MRC Centre for Environment and Health, Imperial College London, London, UK; c Department of Primary Care & Public Health, Imperial College London, London, UK; d Division of Metabolism, Digestion and Reproduction, Imperial College London, London, UK; e Cancer Screening and Prevention Research Group (CSPRG), Department of Surgery and Cancer, Imperial College London, London, UK; f Abdul Latif Jameel Institute for Disease and Emergency Analytics, Imperial College London, London, UK; g Office on Smoking and Health, US Centers for Disease Control and Prevention, Atlanta, GA, USA; h NHS England and NHS Improvement, London, UK; i Department of Diabetes and Endocrinology, St Mary's Hospital, Imperial College Healthcare NHS Trust, London, UK; j Regional Institute for Population Studies, University of Ghana, Accra, Ghana

## Abstract

**Background:**

Diabetes leads to a wide range of established vascular and metabolic complications that has resulted in the implementation of diverse prevention programmes across high-income countries. Diabetes has also been associated with an increased risk of a broader set of conditions including cancers, liver disease, and common infections. We aimed to examine the trends in a broad set of cause-specific hospitalisations in individuals with diabetes in England from 2003 to 2018.

**Methods:**

In this epidemiological analysis, we identified 309 874 individuals 18 years or older with diabetes (type 1 or 2) in England from the Clinical Practice Research Datalink linked to Hospital Episode Statistics inpatient data from 2003 to 2018. We generated a mixed prevalent and incident diabetes study population through serial cross sections and follow-up over time. We used a discretised Poisson regression model to estimate annual cause-specific hospitalisation rates in men and women with diabetes across 17 cause groupings. We generated a 1:1 age-matched and sex-matched population of individuals without diabetes to compare cause-specific hospitalisation rates in those with and without diabetes.

**Findings:**

Hospitalisation rates were higher for all causes in persons with diabetes than in those without diabetes throughout the study period. Diabetes itself and ischaemic heart disease were the leading causes of excess (defined as absolute difference in the rate in the populations with and without diabetes) hospitalisation in 2003. By 2018, non-infectious and non-cancerous respiratory conditions, non-diabetes-related cancers, and ischaemic heart disease were the most common causes of excess hospitalisation across men and women. Hospitalisation rates of people with diabetes declined and causes of hospitalisation changed. Almost all traditional diabetes complication groups (vascular diseases, amputations, and diabetes) decreased, while conditions non-specific to diabetes (cancers, infections, non-infectious and non-cancerous respiratory conditions) increased. These differing trends represented a change in the cause of hospitalisation, such that the traditional diabetes complications accounted for more than 50% of hospitalisation in 2003, but only approximately 30% in 2018. In contrast, the proportion of hospitalisations due to respiratory infections between the same time period increased from 3% to 10% in men and from 4% to 12% in women.

**Interpretations:**

Changes in the composition of excess risk and hospitalisation burden in those with diabetes means that preventative and clinical measures should evolve to reflect the diverse set of causes that are driving persistent excess hospitalisation in those with diabetes.

**Funding:**

Wellcome Trust.

## Introduction

Diabetes is known to lead to a wide range of complications including acute metabolic decompensation such as diabetic ketoacidosis,^1^ macrovascular diseases such as ischaemic heart disease,^2^ stroke,^2^, ^3^ and microvascular complications such as renal disease,^4^ retinopathy, and peripheral neuropathy.^5^ As these traditional complications are largely preventable through evidence-based interventions, they have become the focus of guidelines targeting future events in people with diabetes.

Previous work has found declines in non-fatal incidence of these traditional diabetes complications,^5^ in parallel with declines in all-cause mortality in people diagnosed with diabetes.^6^, ^7^, ^8^ The largest reductions in mortality in this context have been attributed to reductions in macrovascular causes;^6^, ^7^, ^8^ however, the contributing factors are probably mixed.^9^ There is also evidence from both cohort studies and population-based surveillance of mortality that a broad set of non-traditional conditions are accounting for an increasing share of morbidity in people with diabetes.^6^ Emerging non-traditional complications include common infections,^10^ liver disease,^11^ dementia,^12^, ^13^ and a number of site-specific cancers.^14^, ^15^ Although less specific to diabetes, each of these complications has established mechanisms, and consistently shows increased risk of mortality, in people with diabetes. The increased risk of mortality is reflected in findings in 2021 that cancer now accounts for the greatest excess mortality risk between people with and without diabetes.^8^

**Research in context**

**Evidence before this study**

We searched PubMed for reports of population-based analyses of trends in the causes of hospitalisation in people with diabetes from Jan 1, 1990, to April 30, 2021, using the terms “hospitalisation trends” and “diabetes mellitus” along with “cause-specific morbidity” and “diabetes mellitus”. Research to date, predominantly in other countries, suggests that the incidence of traditional diabetes complications such as acute myocardial infarction and stroke has declined in those with diabetes. Trends in other morbidity causes in people with diabetes are unclear.

**Added value of this study**

In this observational analysis of more than 300 000 individuals with diabetes in England, we found substantial differences in hospitalisation trends across conditions. Traditional diabetes complications declined from 2003 to 2018 and hospitalisation rates for other conditions, such as common infections, cancers, and respiratory disease, increased. These trends resulted in a diversification in composition of both the cause of hospitalisation and the composition of excess hospitalisations in people with diabetes, from vascular conditions to cancers and infections. This study provides insight into the drivers of excess and persistent morbidity in individuals with diabetes more comprehensively and holistically than previous studies.

**Implications of all the available evidence**

The composition of excess risk and hospitalisation burden in those with diabetes has transitioned from traditional diabetes-related complications to a broader set of conditions including common infections, cancers, and respiratory diseases. If we are to replicate the improvements in all-cause mortality in those with diabetes in improvements in hospitalisation and morbidity rates, clinical and targeted preventative efforts should broaden to encompass the diversity of excess hospitalisation risk faced by those with diabetes.

Morbidity and hospitalisations of people with diabetes are not only due to diabetes-specific complications, but other conditions that are likely to comprise a large and growing proportion of hospitalisations. These non-specific complications have implications for preventative and clinical approaches to reduce morbidity in this patient group, yet few studies have previously examined them. We therefore aimed to examine the trends in a broad set of cause-specific hospitalisations of individuals with diabetes from 2003 to 2018 in England.

## Methods

### Study design and population

In this epidemiological analysis, we estimated cause-specific hospitalisation rates in 309 874 adults with diagnosed diabetes in England, UK, from Jan 1, 2003, to Oct 30, 2018, using the Clinical Practice Research Datalink (CPRD) linked to Hospital Episode Statistics (HES). 2003 was used as the start year because of the increased linkage and data quality from this year onwards, and because the UK National Health Service (NHS) quality and outcomes framework, which incentivises the identification of individuals with chronic diseases such as diabetes, was introduced in this year. CPRD is a primary care database of over 45 million people registered in 674 general practices in England,^16^ initiated in 1994. The CPRD population is broadly representative of the population in terms of age, sex, and ethnicity.^16^

We extracted records from the CPRD of patients with diabetes in the specified timeframe to generate a mixed prevalent and incident diabetes study population through serial cross sections and follow-up over time. We identified individuals with diabetes (both type 1 and type 2) using both diagnostic (C10) and management (66A) codes from the Read and Oxford Medical Information System for diabetes^17^ and glucose lowering therapy prescription data. We included those with diabetes in the study from the date of diabetes diagnosis if they were 18 years or older (or if they became 18 during study follow-up), if the patient record was marked as acceptable for research by CPRD, and if CPRD deemed that the participating primary care practice was contributing up-to-standard data. Individuals identified as having diagnosed diabetes were included in the study population from the latest of either the event date of a clinically recorded (Read code) diagnosis of diabetes or diabetes-related clinical encounter, the event date of prescription of a glucose lowering therapy, providing that the patient record had at least two such prescriptions, or the study start date (Jan 1, 2003). Individuals in the dataset with diabetes diagnosed before 2003 were therefore included in the study population from Jan 1, 2003. Individuals remained in their respective study populations until either death, leaving CPRD, or the end of the study period (Oct 30, 2018). The CPRD registered study protocol number was 19_118.

### Matched non-diabetes participants

We initially matched the diabetes participants to individuals within the CPRD dataset without diabetes who were the same age (year of birth), same sex, who were present in the dataset from the same year they joined the cohort or earlier, and who were linked to HES data. This provided matches for less than 70% of the diabetes population. Therefore, we matched diabetes participants to people without diabetes by decade of birth rather than year of birth, resulting in 309 874 (100%) matched individuals without diabetes. There were 185 914 individuals without diabetes in the study cohort in 2003, increasing to 217 510 in 2018 (table 1). Individuals in the non-diabetes comparison group were included in that population from the date their record entered into CPRD and CPRD quality checks were confirmed on both their individual and practice record; namely, the patient record was marked as acceptable for research by CPRD, and CPRD deemed that the participating primary care practice was contributing up-to-standard data. Individuals with a diabetes diagnosis were eligible for inclusion in the non-diabetes population until their diagnosis date, at which point they were censored and would enter the diabetes study population.

**Table 1:** Background characteristics of men and women study participants with and without diabetes, 2003–18

		**Diabetes population**				**Non-diabetes population**			
		**2003**	**2007**	**2013**	**2018**	**2003**	**2007**	**2013**	**2018**
**Men**									
Prevalent cohort, n		51 019	78 391	115 251	117 345	100 288	107 606	117 438	114 464
Age, years		64 (29–87)	64 (54–74)	65 (55–76)	67 (57–77)	57 (22–84)	60 (50–71)	63 (53–74)	66 (56–76)
Body-mass index (%)		..	..	..	..	..	..	..	..
	< 25 kg/m^2^	6745 (13·2%)	9771 (12·5%)	10 141 (8·8%)	3164 (2·7%)	461 (0·5%)	3660 (3·4%)	3439 (2·9%)	581 (0·5%)
	25–30 kg/m^2^	14 724 (28·9%)	22 289 (28·4%)	23 234 (20·2%)	6964 (5·9%)	1311 (1·3%)	5607 (5·2%)	4938 (4·2%)	719 (0·6%)
	>30 kg/m^2^	13 529 (26·5%)	24 826 (31·7%)	29 456 (25·6%)	8588 (7·3%)	1271 (1·3%)	3433 (3·2%)	3133 (2·7%)	393 (0·3%)
	No BMI measurement	16 021 (31·4%)	21 505 (27·4%)	52 420 (45·5%)	98 629 (84·1%)	97 245 (97·0%)	94 906 (88·2%)	105 928 (90·2%)	112 771 (98·5%)
Age at time of diabetes diagnosis, years		58 (23–82)	57 (47–67)	56 (47–66)	54 (45–64)	NA	NA	NA	NA
Diabetes duration		..	..	..	..	..	..	..	..
	0–2 years	18 188 (35·6%)	14 098 (18·0%)	13 224 (11·5%)	3824 (3·3%)	NA	NA	NA	NA
	2–5 years	11 866 (23·3%)	20 479 (26·1%)	21 218 (18·4%)	11 663 (9·9%)	NA	NA	NA	NA
	5–10 years	11 459 (22·5%)	23 717 (30·3%)	35 173 (30·5%)	32 685 (27·9%)	NA	NA	NA	NA
	10–20 years	8825 (17·3%)	17 784 (22·7%)	36 853 (32·0%)	53 486 (45·6%)	NA	NA	NA	NA
	>20 years	681 (1·3%)	2313 (3·0%)	8783 (7·6%)	15 687 (13·4%)	NA	NA	NA	NA
**Women**									
Prevalent cohort, n		43 841	68 596	99 754	102 202	85 626	93 947	105 569	103 046
Age, years		68 (26–91)	67 (55–80)	66 (53–79)	66 (53–79)	60 (16–89)	61 (48–74)	64 (50–78)	66 (53–80)
Body-mass index (%)		..	..	..	..	..	..	..	..
	< 25 kg/m^2^	5955 (13·6%)	9141 (13·3%)	8995 (9·0%)	2651 (2·6%)	316 (0·4%)	4233 (4·5%)	3901 (3·7%)	536 (0·5%)
	25–30 kg/m^2^	9119 (20·8%)	13961 (20·4%)	14431 (14·5%)	4196 (4·1%)	650 (0·8%)	3528 (3·8%)	3085 (2·9%)	309 (0·3%)
	>30 kg/m^2^	13 872 (31·6%)	24 421 (35·6%)	27425 (27·5%)	8223 (8·0%)	1014 (1·2%)	3219 (3·4%)	2735 (2·6%)	189 (0·2%)
	No BMI measurement	14 895 (34·0%)	21 073 (30·7%)	48 903 (49·0%)	87 132 (85·3%)	83 646 (97·7%)	82 976 (88·3%)	95 848 (90·8%)	102 012 (99·0%)
Age at time of diabetes diagnosis, years		61 (22–86)	60 (48–72)	57 (45–70)	54 (42–67)	NA	NA	NA	NA
Diabetes duration		..	..	..	..	..	..	..	..
	0–2 years	16 170 (36·9%)	12 995 (18·9%)	11 810 (11·8%)	3467 (3·4%)	NA	NA	NA	NA
	2–5 years	10 068 (23·0%)	18 781 (27·4%)	18 375 (18·4%)	10 993 (10·8%)	NA	NA	NA	NA
	5–10 years	9 597 (21·9%)	20 229 (29·5%)	31 509 (31·6%)	28 471 (27·9%)	NA	NA	NA	NA
	10–20 years	7473 (17·0%)	14 770 (21·5%)	30 846 (30·9%)	46 659 (45·7%)	NA	NA	NA	NA
	>20 years	533 (1·2%)	1821 (2·7%)	7214 (7·2%)	12 612 (12·3%)	NA	NA	NA	NA

Data are n (%) or median (IQR). Missingness of body-mass index measurements annually is variable over time and across diabetes and non-diabetes populations. NA=not applicable.

We used the HES admitted inpatient linked dataset and a previously used grouping approach to collapse any potential multiple hospital episodes^18^, ^19^ into one hospital spell (identified by a unique hospital spell number for each patient's admission) so that each person admitted to hospital was counted in our dataset only once for each inpatient spell irrespective of how many times they were transferred between physicians and hospitals. If there were multiple primary diagnoses matching our cause groupings within one spell we used the first diagnosis within the spell. We identified and removed admissions identified as regular (ie, planned attendances for chemotherapy or dialysis).

To examine the spectrum of causes comprising the hospitalisation burden we included several categories of morbidity: traditional diabetes-specific complications with established specific relationships to diabetes (including acute metabolic decompensations [eg, hyperglycaemic crisis], cardiovascular diseases [eg, acute myocardial infarction], microvascular disease effects [eg, chronic kidney disease]); non-specific diabetes complications (ie, conditions with increased risk for those with diabetes, including liver disease, site-specific cancers [namely colorectal, pancreatic, liver, breast, endometrial, and gallbladder], and common infections); and other associated conditions that have not traditionally been considered either classic microvascular or macrovascular complications, nor have emerging associations, but which previous analyses have shown to have a high attributable portion of mortality burden in the diabetes population (eg, respiratory disease, all other cancers).^8^

There were 17 cause-specific hospitalisation groupings included in the final analysis; namely, acute myocardial infarction; other ischaemic heart disease (including heart failure); stroke; diabetes (admission underlying diagnosis coded as non-hyperglycaemic crisis diabetes); hyperglycaemic crisis; major amputations; minor amputations; diabetes-related cancer (cancers with strong evidence of causal association); all other (non-diabetes-related) cancers; respiratory infections; kidney infections; skin and bone infections; sepsis; chronic kidney disease; other renal disease; other respiratory disease; and liver disease. We further grouped these into seven broader cause groupings: vascular (excluding amputations); cancer; infections; amputations (and skin or bone infections); diabetes; respiratory, renal, and liver conditions; and sepsis. The International Classification of Diseases (ICD)-10 definitions for each cause groupings, including underlying causes are outlined in the appendix (pp 1–2).

### Statistical analyses and outcomes

We examined year (period), age, diabetes duration (set to 0 in individuals without diabetes), and death status as time-dependent variables. Diabetes status (yes or no) and sex (male or female) were non-time-dependent variables. We used a discrete Poisson regression model^17^ to estimate annual cause-specific hospitalisation rates in the diabetes population. We split the study period into discrete year intervals. Each individual was allocated an exposure length for each discrete year ranging between 0 and 1 (with the exception of the final study year 2018 for which maximum exposure was 0·83 years). Each individual had a cause-specific exposure for each discrete year they were in the study population. For example, an individual who had a hospitalisation with acute myocardial infarction as the underlying cause on June 30, 2014, their corresponding acute myocardial infarction exposure period (denominator) for 2014 would be 0·5 years (6 months). The age, diabetes duration, and death status of each individual were updated for each discrete year period.

We used the first hospitalisation of each cause-specific hospitalisation per calendar year (secondary admissions for the same condition in the same calendar year were not used). For example, for an individual who had two hospitalisations with stroke as the underlying cause in 2011, one on Jan 31 and a second on Aug 24, their first hospital admission would be counted in the numerator for hospitalisation rates in that year, and their corresponding stroke exposure period (denominator) for 2011 would be 0·08 years (1 month). For an individual who had a hospitalisation for acute myocardial infarction on Feb 28, 2013, for cancer on June 30, 2013, and for and respiratory infection on September 30, 2013, their exposure length for each cause-specific hospitalisation calculation would be 0·17 years (2 months), 0·5 years (6 months), and 0·75 years (9 months), respectively (appendix p 7).

We examined the observed, unadjusted, cause-specific hospitalisation rates to assess trends for linearity visually. For the causes that had linear trends we used time (calendar year) as a continuous variable, and treated follow-up time as a single time period (non-piecewise approach). For the causes with non-linear trends we applied a piecewise approach whereby we split the time into four 4-year periods: 2003–06, 2007–10, 2011–14, and 2015–18. We used the marginal rate from the Poisson regression to estimate age-adjusted all-cause and cause-specific hospitalisation rates per 10 000 people. For the sex-specific analysis we used separate (stratified) models for men and women. This model included interaction terms between diabetes status and discrete year and age. Because we adjusted for age of the entire study population, the reported hospitalisation rates for each year in the study period (2003–18) correspond to a population that has the same age distribution as the entire sample, those with and without diabetes, over the entire analysis period for men and women respectively. Excess hospitalisation rates between the diabetes and non-diabetes populations were calculated as the absolute difference in adjusted rates in a given year. For age-specific analyses, we stratified by three age groups (<55, 55–75, >75 years). We used R Studio version 1.2.5042 for all data management and for the assembly of datasets, and Stata version 16.1 for the statistical analysis.

### Role of the funding source

The funder of the study had no role in study design, data collection, data analysis, interpretation of data, or writing of the report.

## Results

Of the individuals included in the study, there were approximately 51 000 men and 44 000 women in the diabetes population in 2003, and 117 000 men and 102 000 women in 2018. Individuals with diabetes were sex-matched with people without diabetes. At the beginning of the study period, in the diabetes population, the median age of men was 64 years (IQR 29-87) and of women was 68 years (IQR 26-91), whereas in the non-diabetes population the median age of men was 57 (IQR 22-84) and of women it was 60 (IQR 16-89) in 2003. Over the 16-year study period, the median age of men in the diabetes population and the non-diabetes population increased and by the end of the study period the differences in median ages between the diabetes and non-diabetes populations were eliminated in both sexes (table 1). In the diabetes population, the prevalence of obesity increased over time but decreased in the population without diabetes. The diabetes duration also increased over time in the diabetes population, with participants who had had diabetes for over 20 years making up 1·2% of the diabetes group (533 men and 681 women) in 2003, increasing to 12612 (12%) women with diabetes and 15687 (13%) men with diabetes by 2018 (table 1).

**Figure 1:**
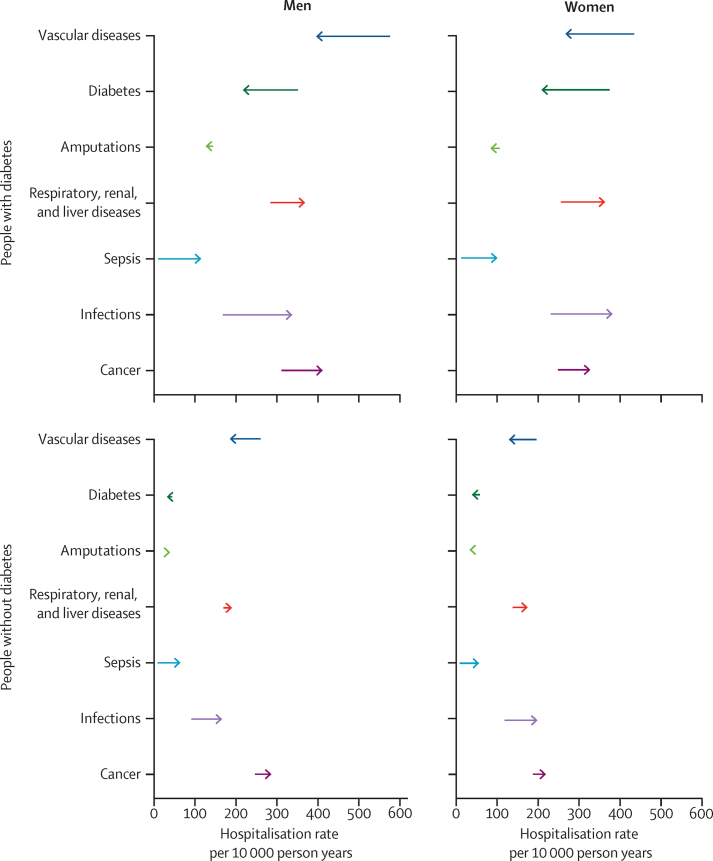
Absolute change in adjusted hospitalisation rates by cause grouping from 2003 to 2018 in those with and without diabetes The ends of the arrow tails represent hospitalisation rates in 2003 and the tips of the arrow heads represent hospitalisation rates in 2018.

Hospitalisation rates for all causes studied were higher in people with diabetes than in those without diabetes at the beginning and end of our study period (table 2; figure 1). Excess hospitalisation rates (the absolute difference in adjusted hospitalisation rates between people with and without diabetes) varied by sex and time. In 2003, in men with diabetes, hospitilisation for diabetes itself (non-hyperglycaemic crisis) was the leading cause of excess hospitalisation (an excess of 158·0 hospitalisations per 10 000 men) followed by ischaemic heart disease (138·9 excess hospitalisations per 10 000 men) and non-infectious, non-cancerous respiratory conditions (49·5 excess hospitalisations per 10 000 men). In 2018, ischaemic heart disease was the leading cause of excess hospitalisation (98·7 excess hospitalisations per 10 000 men) followed by non-diabetes-related cancers (89·7 excess hospitalisations per 10 000 men) and respiratory infections (75·8 excess hospitalisations per 10 000 men). In women, diabetes (159·4 excess hospitalisations per 10 000 women) and ischaemic heart disease (87·9 excess hospitalisations per 10 000 women) were also the leading causes of excess hospitalisation in 2003, followed by kidney infections (64·7 excess hospitalisations per 10 000 women). However, in 2018, these previously leading causes of excess hospitalisations in women were no longer in the top three causes of excess hospitalisation, having been replaced by non-infectious and non-cancerous respiratory conditions (93·5 excess hospitalisations per 10 000 women), respiratory infections (76·3 excess hospitalisations per 10 000 women), and non-diabetes-related cancers (63·6 excess hospitalisations per 10 000 women; table 2; figure 1).

**Table 2:** Adjusted cause-specific and excess hospitalisation (absolute difference) rates per 10 000 person-years in 2003 and 2018 in men and women with and without diabetes

		**2003**			**2018**		
		Hospitalisations in the diabetes population	Hospitalisations in the non-diabetes population	Excess hospitalisation	Hospitalisations in the diabetes population	Hospitalisations in the non-diabetes population	Excess hospitalisation
**Men**							
Vascular diseases		..	..	..	..	..	..
	Ischemic heart disease	278·4 (269·0–287·9)	139·5 (134·3–144·5)	138·9	197·8 (192·6–203·1)	99·1 (96·3–101·9)	98·7
	Stroke	70·6 (66·1–75·2)	46·1 (43·0–49·2)	24·5	55·0 (52·4–57·7)	35·9 (34·2–37·7)	19·1
	Acute myocardial infarction	78·2 (73·3–83·1)	45·5 (42·5–48·6)	32·6	59·2 (56·4–62·0)	34·5 (32·8–36·2)	24·7
Cancers		..	..	..	..	..	..
	Diabetes-related cancers	46·3 (43·8–48·8)	25·8 (24·1–27·4)	20·5	44·9 (42·6–47·3)	20·2 (19·0–21·4)	24·7
	Non-diabetes-related cancers	238·9 (233·6–244·3)	197·8 (193·2–202·3)	41·2	335·4 (328·7–342·0)	245·7 (241·0–250·4)	89·7
	Diabetes	..	..	..	..	..	..
Diabetes		165·1 (156·5–173·8)	7·1 (6·3–7·9)	158·0	75·5 (72·3–78·8)	3·2 (2·9–3·6)	72·3
	Hyperglycaemic crisis	54·4 (50·1–58·6)	20·6 (18·8–22·3)	33·8	76·0 (72·8–79·2)	28·8 (27·2–30·3)	47·2
Amputations		..	..	..	..	..	..
	Major Amputations	16·9 (14·1–19·7)	0·5 (0·3–0·8)	16·4	6·2 (5·4–7·0)	0·2 (0·1–0·3)	6·0
	Minor Amputations	20·9 (17·9–24·0)	0·3 (0·2–0·5)	20·6	14·4 (13·1–15·6)	0·2 (0·1–0·3)	14·2
	Skin and bone infections	57·6 (55·1–60·1)	23·8 (22·1–25·6)	33·8	66·6 (64·2–69·0)	35·9 (33·7–38·1)	30·7
Infections		..	..	..	..	..	..
Respiratory infections		64·6 (62·1–67·0)	44·3 (42·3–46·4)	20·2	172·4 (167·6–177·2)	96·7 (93·5–99·8)	75·8
	Kidney infections	75·4 (72·6–78·2)	38·0 (36·0–40·0)	37·4	108·4 (105·0–111·8)	54·5 (52·2–56·7)	53·9
	Sepsis	13·5 (11·6–15·5)	6·7 (5·7–7·8)	6·8	115·6 (110·5–120·7)	57·5 (54·7–60·3)	58·1
Respiratory, renal, and liver diseases		..	..	..	..	..	..
	Respiratory disease (non-infectious, non-cancerous)	162·1 (157·7–166·6)	112·6 (109·0–116·2)	49·5	179·3 (174·8–183·8)	114·3 (111·1–117·5)	65·0
	Renal disease	59·0 (56·6–61·4)	34·3 (32·4–36·2)	24·7	102·0 (98·7–105·4)	49·6 (47·3–51·8)	52·5
	Chronic kidney disease	21·9 (20·4–23·5)	13·6 (11·7–15·6)	8·3	18·0 (17·0–19·0)	10·1 (8·8–11·4)	7·9
	Liver disease	18·2 (15·8–20·6)	5·6 (4·8–6·4)	12·6	25·9 (23·6–28·1)	8·0 (7·2–8·7)	17·9
**Women**							
Vascular diseases		..	..	..	..	..	..
	Ischemic heart disease	179·8 (171·7–187·9)	92·0 (87·4–96·5)	87·9	118·3 (114·0–122·5)	60·5 (58·2–62·8)	57·8
	Stroke	69·1 (64·4–73·9)	45·4 (42·1–48·7)	23·8	53·5 (50·7–56·4)	35·1 (33·3–37·0)	18·4
	Acute myocardial infarction	54·2 (49·8–58·6)	31·5 (28·7–34·3)	22·7	33·9 (31·7–36·1)	19·7 (18·3–21·1)	14·2
Cancers		..	..	..	..	..	..
	Diabetes-related cancers	75·1 (71·7–78·5)	46·9 (44·7–49·1)	28·2	80·9 (77·3–84·5)	50·4 (48·3–52·6)	30·5
	Non-diabetes-related cancers	159·3 (154·7–163·9)	135·8 (132·0–139·7)	23·5	229·7 (223·7–235·7)	166·1 (162·0–170·3)	63·6
Diabetes		..	..	..	..	..	..
	Diabetes	164·3 (154·6–174·0)	4·9 (4·2–5·6)	159·4	57·3 (54·5–60·0)	1·7 (1·5–2·0)	55·6
	Hyperglycaemic crisis	74·9 (69·6–80·2)	28·8 (26·6–31·0)	46·1	94·7 (90·8–98·6)	36·4 (34·6–38·2)	58·3
Amputations		..	..	..	..	..	..
	Major Amputations	9·3 (7·0–11·6)	0·3 (0·1–0·5)	9·0	2·1 (1·7–2·6)	0·1 (0·0–0·1)	2·1
	Minor Amputations	10·4 (8·1–12·8)	0·3 (0·1–0·5)	10·1	5·2 (4·4–6·0)	0·1 (0·1–0·2)	5·0
	Skin and bone infections	55·1 (52·4–57·7)	25·6 (23·6–27·5)	29·5	52·2 (49·8–54·5)	30·0 (28·1–32·0)	22·1
Infections		..	..	..	..	..	..
	Respiratory infections	59·4 (57·0–61·8)	38·4 (36·4–40·4)	21·0	174·0 (168·6–179·4)	97·7 (94·2–101·2)	76·3
	Kidney infections	133·8 (129·8–137·9)	69·2 (66·3–72·1)	64·7	144·1 (140·1–148·2)	83·1 (80·1–86·1)	61·0
	Sepsis	13·8 (11·7–15·9)	6·6 (5·6–7·6)	7·2	103·7 (98·5–108·8)	49·7 (46·9–52·5)	54·0
Respiratory, renal, and liver diseases		..	..	..	..	..	..
	Respiratory disease (non-infectious, non-cancerous)	163·0 (158·3–167·7)	100·4 (97·0–103·8)	62·6	204·4 (199·0–209·8)	110·9 (107·5–114·3)	93·5
	Renal disease	43·9 (41·8–46·0)	18·7 (17·3–20·1)	25·2	82·2 (79·1–85·4)	40·8 (38·5–43·2)	41·4
	Chronic kidney disease	19·4 (17·8–21·1)	7·3 (5·8–8·7)	12·1	13·0 (12·1–13·9)	4·9 (3·9–5·8)	8·1
	Liver disease	9·7 (8·8–11·6)	3·3 (2·7–4·0)	6·4	18·2 (16·3–20·2)	6·3 (5·6–7·0)	11·9

Data are rate per 10 000 (95% CI) or rate per 10 000. Condition groups are mutually exclusive (appendix p 1–2; eg, respiratory includes all respiratory diseases excluding respiratory infections and cancers). The reported rates in 2003 and 2018 correspond to a population that has the same age distribution as the entire sample, those with and without diabetes, over the entire analysis period.

**Figure 2:**
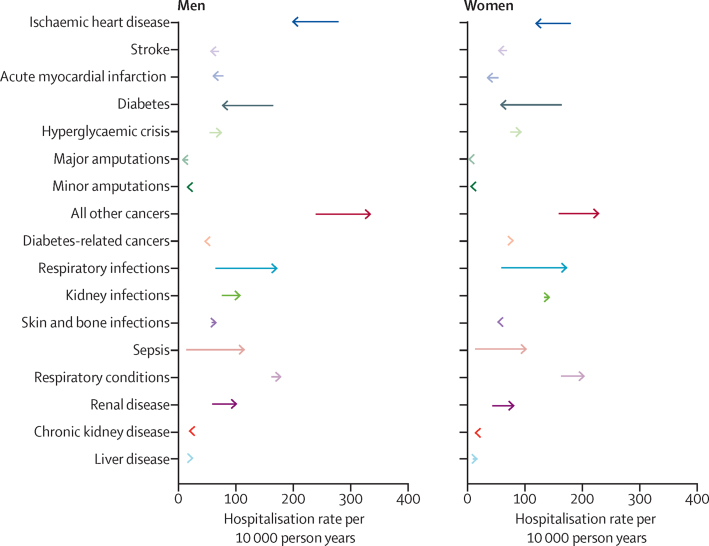
Absolute change in adjusted cause-specific hospitalisation rates from 2003 to 2018 in men and women with diabetes The ends of the arrow tails represent hospitalisation rates in 2003 and the tips of the arrow heads represent hospitalisation rates in 2018.

There was substantial variation in trends of hospitalisation rates by cause grouping. Hospitalisation rates due to vascular disease, diabetes, and amputations declined between 2003 and 2018, and hospitalisation rates due to cancers not related to diabetes, respiratory infections, renal infections, sepsis, and renal, respiratory, and liver disease increased between 2003 and 2018 (figure 2). This variation in trends was generally similar across men and women, with higher rates of hospitalisation in vascular, non-diabetes-related cancer, and amputations in men than in women throughout the study (table 2). The largest declines in the rates of hospitalisations between 2003 and 2018 were seen in ischaemic heart disease, which declined by 29·0% in men (from 278·4 hospitalisations to 197·8 hospitalisations per 10 000 men) and 34·2% in women (from 179·8 hospitalisations to 118·3 hospitalisations per 10 000 women), and diabetes (non-hyperglycaemic crisis), which declined by 54·3% in men (from 165·1 hospitalisations to 75·5 hospitalisations per 10 000 men) and 65·1% in women (from 164·3 hospitalisations to 57·3 hospitalisations per 10 000 women) (table 2). The magnitude of declines in rates of hospitalisations over the study period was more modest for acute myocardial infarction and stroke than it was for ischaemic heart disease and (non-hyperglycaemic crisis) diabetes (table 2; figure 2). In contrast to all other traditional diabetes complications, hospitalisation for hyperglycaemic crises increased by 39·7% in men (54·4 hospitalisations to 76·0 hospitalisations per 10 000 men) and by 26·4% in women (74·9 hospitalisations to 94·7 hospitalisations per 10 000 women) (table 2). Over the study period, hospitalisation rates for diabetes-related cancers remained stable (whereas there was a large increase in hospitalisations due to all other cancers) and declines in hospitalisations due to major amputations was much greater than the declines seen in hospitalisations due to minor amputations. The largest observed increases in hospitalisations between 2003 and 2018 were due to non-diabetes-related cancers (an increase of 40·4% [238·9 hospitalisations to 335·4 hospitalisations per 10 000 men] and 44·2% [159·3 hospitalisations to 229·7 hospitalisations per 10 000 women]) and respiratory infections (an increase of 166·9% [64·6 hospitalisations to 172·4 hospitalisations per 10 000 men] and 192·9% [59·4 hospitalisations to 174·0 hospitalisations per 10 000 women]) (table 2). Hospitalisations for renal disease also increased substantially (59·0 hospitalisations to 102·0 hospitalisations per 10 000 men and 43·9 hospitalisations to 82·2 hospitalisations per 10 000 women). Similar trends were observed across age groups (appendix p 8).

**Figure 3:**
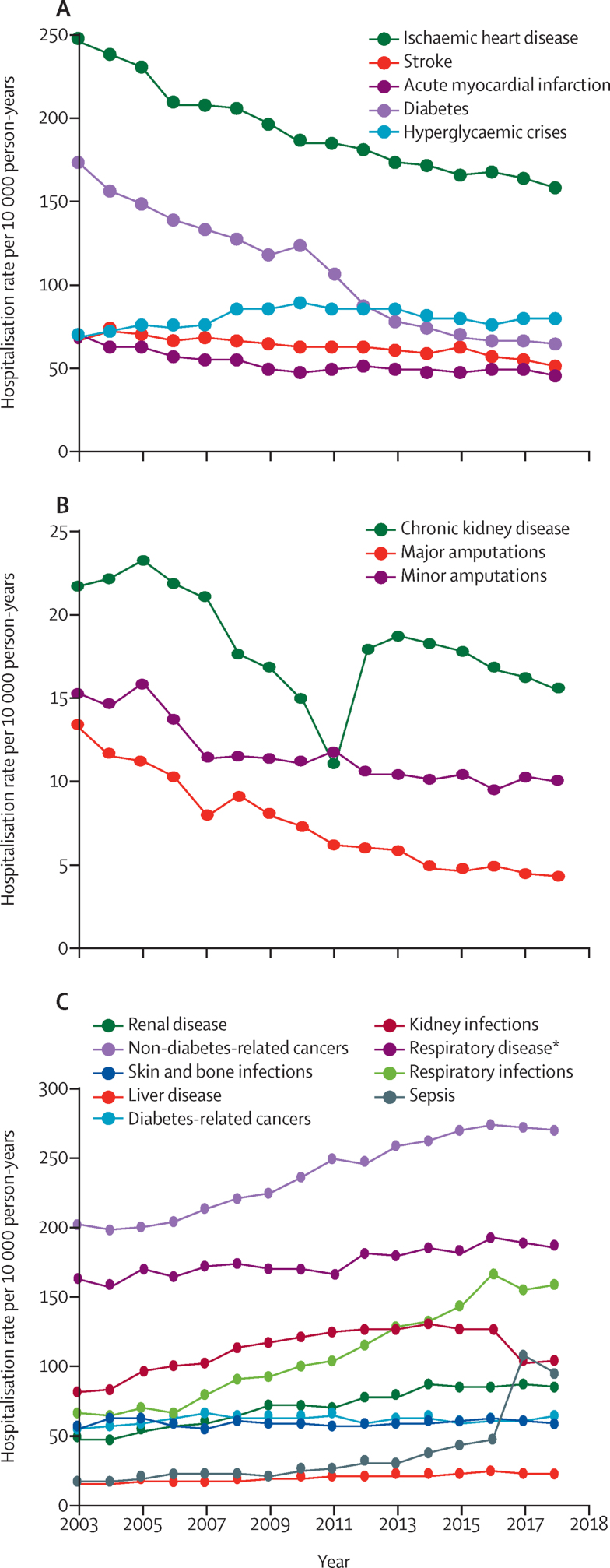
Hospitalisation rates due to diabetes-specific complications (A and B) and other causes (C) in men and women with diabetes from 2003 to 2018 *Non-infectious, non-cancerous.

Although the hospitalisation rates for diabetes-specific complications declined between 2003 and 2018, the pace of decline in hospitalisations due to ischaemic heart disease, diabetes, acute myocardial infarction, stroke, and major amputations diminished in the latter years (figure 3A, 3B). The rate of hospitalisations due to minor amputations only declined slightly after 2007 and the rate of hospitalisations due to chronic kidney disease declined steadily from 2005 to 2011, after which there was a much flatter decline between 2012 and 2018 (with a notable negative spike in 2011). Trends in hospitalisation rates for non-specific-diabetes complications were generally more uniform throughout the period, with the largest increases in rates of hospitalisations observed due to all other cancers, respiratory infections, and sepsis; the latter seeing a pronounced increase in 2017–18 (figure 3C).

**Figure 4:**
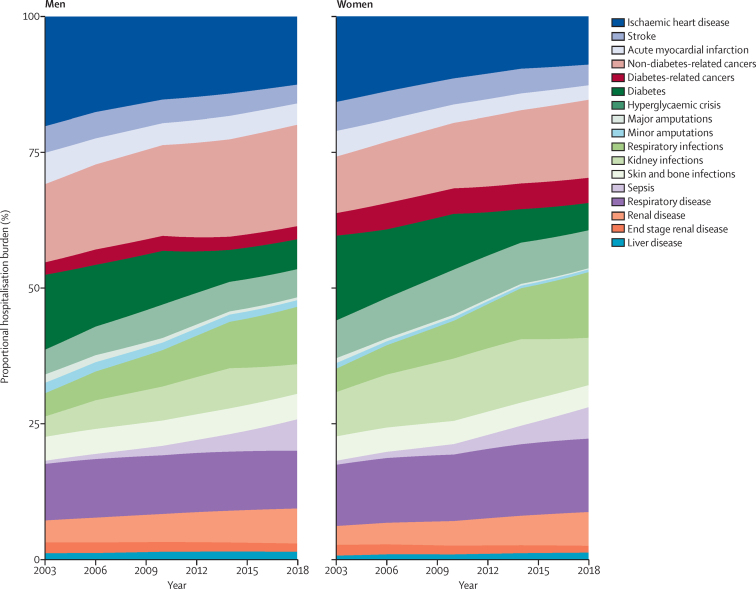
Proportional contribution to hospitalisation burden of leading cause-specific diseases in people with diabetes from 2003 to 2018

The heterogenous trends across conditions contributed to a diversification in causes of hospitalisation over the 16-year period, away from diabetes-specific complications. The proportion of hospitalisations due to vascular diseases declined from 31% to 20% in men and from 28% to 16% in women from 2003 to 2018 (figure 4; appendix pp 3–6). The three traditional diabetes-specific complication groupings of vascular, diabetes, and amputations collectively accounted for around half of the hospitalisation burden of all of our causes in 2003 (58% in men and 49% in women), declining to close to one-third in 2018 (38% in men and 28% in women). Conversely, the proportion of hospitalisations due to cancer increased from 17% in 2003 to 21% in 2018 in men and from 16% in 2003 to 20% in 2018 in women, with much larger increases in hospitalisations due to non-diabetes-related cancers than diabetes-related cancers (figure 4; appendix pp 3–6). The largest increase in the proportion of hospitalisations that a cause is attributal for was seen in the infections grouping, in which hospitalisations due to respiratory infections increased from 4·3% to 10·6% in men and from 4·6% to 12·4% in women. Hospitalisations due to sepsis increased from 1% to 6% in both men and women.

## Discussion

In this comprehensive examination of leading causes of hospitalisations in people with diabetes, we found stark differences in the long-term trends in hospitalisation for traditional diabetes complications and other forms of morbidity in those with diabetes. There are four particularly noteworthy trends. There was a gradual transition in causes of hospitalisation from traditional diabetes-related complications to a more diverse set of conditions that are not specific to diabetes, including common infections such as respiratory and kidney infections and non-diabetes-related cancers. Hospitalisation rates for traditional diabetes complications (vascular disease, amputations, and diabetes) declined from 60% of all hospitlisations studied in 2013 to 30% in 2018, and hospitalisation rates for other leading causes of hospitalisation including cancers, respiratory and renal infections, and respiratory conditions increased over the same period.

This trend is further illustrated by non-infectious and non-cancerous respiratory diseases becoming the leading cause of hospitalisations in women with diabetes in 2018. The specific preventative efforts targeting traditional diabetes complications have helped reduce both mortality and hospitalisations, and this has contributed to a more diverse set of hospitalisation causes, all of which remain at a higher level in the diabetes population than in the matched population without diabetes.

Second, the large declines in hospitalisation rates in people with diabetes for cardiovascular conditions, diabetes, and amputations in our study are consistent with trends in diabetes complications in the USA^5^ and trends in cause-specific mortality^6^ in England^8^ and Australia.^6^, ^20^ Further, our estimates are broadly consistent with the incident rates of complications found in the National Diabetes Audit;^21^ however, our findings include a longer time period and cover more non-traditional diabetes complications. This decline in hospitalisation rates for cardiovascular disease, diabetes, and amputations is likely to be a result of efforts ensuring timely and comprehensive outpatient and community care to reduce unplanned hospital admissions. There are, however, substantial differences in the magnitude of the relative declines in hospitalisation rates for difference causes in the diabetes population of our study. Although hospitalisation rates for cardiovascular conditions declined by between 20% and 30% in this population, the largest declines in hospitalisations were seen in major amputations (declines of between 65% and 75%) and diabetes itself (non-hyperglycaemic crises, including hypoglycaemia; 55–65%). Meanwhile, hospitalisation rates for hyperglycaemic crises actually increased. The more modest decline in minor amputations than the decline in major amputations might reflect earlier detection of diabetes and hence the increased prevention of major surgery. These declines in diabetes-specific conditions might reflect a combination of improvements of various risk factors in England, such as salt intake and hypertension,^22^ as well as specific, targeted, comprehensive prevention measures to avoid future events in this patient group (eg, financial incentives such as the quality and outcomes framework^23^) that focus on controlling such cardiometabolic risk factors.^2^ However, the apparent slowing of improvements in rates of hospitalisation due to cardiovascular disease in the latter years of the study is concerning and consistent with other studies in individuals with diabetes in the USA.^25^

Third, the diversification of the hospitalisation burden over the study period has led to the largest relative increases in hospitalisation being due to respiratory infections in women (192·9% [59·4 hospitalisations to 174·0 hospitalisations per 10 000 women]), renal disease in women (87·2% [43·9 hospitalisations to 82·2 hospitalisations per 10 000 women]), non-diabetes-related cancers in women (44·2% [159·3 hospitalisations to 229·7 hospitalisations per 10 000 women]), and sepsis in women (651·4% [13·8 hospitalisations to 103·7 hospitalisations per 10 000 women]), with the largest increases observed in older adults. The leading cause of hospitalisation transitioned over the study period from ischaemic heart disease to non-diabetes-related cancers in middle aged and elderly individuals, whereas diabetes itself remained the leading cause of hospitalisation in younger adults throughout the study period. The direction of these trends was similar in the non-diabetes population, suggestive that those with diabetes are generally experiencing a wider range of cause-specific hospitalisations than previously. Three potential contributors to this diversification could be declines in competing risks from diabetes-specific complications, such as vascular disease, that have seen the largest declines in hospitalisation rate over the period studied; increased longevity due to an ageing diabetes population with increased duration of disease (age itself is a leading driver of multimorbidity; multimorbidity has increased substantially over the study period^26^ and is a leading predictor in increased hospital admissions seen in the general population^27^); and changing incidence rates of these non-specific diabetes complications, many of which have overlapping risk factors with diabetes.^28^ In contrast to the increase in hospitalisations seen across most infection groups, the approximate halving in hospitalisations due to skin and bone infections might be due to the increased access to multidisciplinary footcare teams for those with diabetic foot disease over this period.

Finally, our findings of observed trends in hospitalisation rates are susceptible to changes in practitioner behaviour, diagnostic criteria, and coding changes. Hospital admissions due to diabetes declined substantially over the study period, and hyperglycaemic hospital admissions increased. Some of this observed trend could reflect increased specificity of diagnostic coding during this period, from a broad diagnosis of diabetes to a more specific cause of hospitalisation (eg, hyperglycaemic crisis, acute myocardial infarction, or infection). Given the substantial decline in mortality attributable to diabetes (including hyperglycaemic crisis) in this patient group over a similar time period, and improved specificity of diagnostic coding,^29^ this explanation is plausible.^8^ Recent initiatives to increase the awareness and identification of sepsis across clinical practice has contributed to the large increase in observed hospitalisation rates for this condition, and our finding of an isolated spike in chronic kidney disease hospital admissions in 2012, following a gradual decline from 2003 to 2011, suggests a change in coding or admission practice for this patient group.

Our findings are consistent with previous work estimating trends in incidences of traditional diabetes complications in other countries. In people in the USA with diabetes, acute myocardial infarction decreased by 68%, stroke by 53%, and amputations by 52% from 1990 to 2010,^5^ although declines appear to have plateaued in younger adults since 2010.^25^ Contrastingly, hospitalisation rates for hyperglycaemic crises^30^ and lower respiratory disease have increased since the 1990s in those with diabetes in the USA.^31^ The incidence and hospitalisation rates for vascular conditions have declined across the general population in England over the time period of our study, with a 33% decline in acute myocardial infarction incidence between 2002–2010 in men and a 31% decline in women.^32^ There have been 5% annual declines in acute myocardial infarction and angina in people with diabetes over a similar time period in England.^33^ Meanwhile, our findings are consistent with previous work finding emergency hospital admissions in England have increased by 42% from 2006 to 2018 (averaging a 3·2% increase per year), with the largest increases in those aged 85 years or older and those with five or more chronic conditions.^27^

Our study uses primary care records linked to hospitalisation data for more than 300 000 individuals with diabetes. This scale enabled us to estimate hospitalisations in great detail with regards to cause, over time, by age, and by sex. CPRD has been used extensively for epidemiological research for many years^16^ and is representative of the English population according to age and sex; however, there are limitations to such administrative datasets and our study. Miscoding, misdiagnosis, and misclassification in administrative datasets such as CPRD has been extensively assessed and documented in previous work.^34^ We therefore use a pragmatic approach to identifying individuals with diabetes within CPRD that considers the combination of diagnostic codes, administrative codes, and medications, overcomes conflicting recordings, and uses a previously described approach for identifying those with diabetes within CPRD,^35^ rather than relying upon specific Read codes alone. Our findings therefore pertain to the population with diagnosed diabetes and this approach does not capture those with undiagnosed diabetes, which might account for approximately 20% of all people with diabetes in the UK.^36^ Further, we did not assess the effect of diabetes-specific policies over the study period including the change in diagnostic criteria from fasting plasma glucose to HbA1C. Additionally, we did not stratify by diabetes type (1 or 2) because we did not have the data to accurately distinguish type 1 from type 2 diabetes. Although our analysis is deliberately broad in causes in scope, it is not comprehensive.

We accounted for several covariates that probably influence hospitalisation risk including age, sex, year, and, advancing on previous studies, duration of diabetes. The non-diabetes population is generally younger than the diabetes population in the earlier years of the study, with the gap in age declining during the study period; our Poisson model adjusts for age to account for this. In our dataset and other administrative datasets, the missingness of risk factor data (eg, BMI measurements or smoking status) is relatively high, particularly in the earlier years of our study in which, for example, approximately only 60% of individuals in CPRD have blood pressure or smoking recorded for the past 3 years, and even less for annual measurements. Corresponding figures for BMI are even lower than blood pressure or smoking at 30%,^16^ and missingness of BMI data improved across the general UK population and most other high-income countries over the time period of our study.^37^ This level of, and probable bias due to, missingness makes it difficult to include such potential confounders in our analysis. Similarly, we were not able to match for CPRD practice, nor match for ethnicity or socioeconomic status as we did for sex and decade of birth. Finally, diagnostic and coding practices over the 16-year period might have affected the apportioning of conditions to be primary or secondary diagnoses during a hospital admission; however, the accuracy of primary diagnoses increased substantially to approximately 96% after the introduction of payment by results in 2002.^29^

Hospitalisation rates of traditional diabetes-specific complications have declined in people with diabetes over the past 16 years, and rates in non-specific diabetes complications including non-diabetes-related cancers, respiratory diseases, and common infections have increased. If we are to replicate the improvements in all-cause mortality and in hospitalisation and morbidity rates, clinical and targeted preventative efforts should broaden to encompass the diversity of excess hospitalisation risk faced by those with diabetes.

## Data sharing

Raw data from this study are stored by the Clinical Practice Research Datalink (CPRD) and can be accessed on successful application to the CPRD.

## Declaration of interests

JP-S reports personal fees from Novo Nordisk A/S, is a partner at Lane Clark & Peacock, and is vice chairman of the Royal Society for Public Health. ME reports personal fees from Prudential, Scor, and Third Bridge, and a charitable grant from the AstraZeneca Youth Health Programme. All other authors report no competing interests.
