# Supplementary material for: Trends in leading causes of hospitalisation of adults with diabetes in England from 2003 to 2018: an epidemiological analysis of linked primary care records
Source: Lancet Diabetes Endocrinol. 2022 Jan;10(1):46–57. doi: 10.1016/S2213-8587(21)00288-6 (PMC8672063; doi:10.1016/S2213-8587(21)00288-6)

THE LANCET
Diabetes & Endocrinology

Supplementary appendix

This appendix formed part of the original submission and has been peer reviewed. We post it as supplied by the authors.

Supplement to: Pearson-Stuttard J, Cheng Y J, Bennett J, et al. Trends in leading causes of hospitalisation of adults with diabetes in England from 2003 to 2018: an epidemiological analysis of linked primary care records. *Lancet Diabetes Endocrinol* 2021; published online Nov 30. http://dx.doi.org/10.1016/S2213-8587(21)00288-6.

Supplemental Table 1. ICD-10 codes for each underlying cause of hospitalisation and cause groupings

Underlying causes	ICD-10 Codes	Tier 1 cause groupings	Tier 2 Cause groupings
Malignant neoplasm of liver and intrahepatic bile ducts	C22	Cancers	DM-related cancers
Malignant neoplasm of colon, rectosigmoid junction and rectum (i.e.colorectal)	C18, C19, C20	Cancers	DM-related cancers
Malignant neoplasms of digestive organs except liver and intrahepatic bile ducts or colorectal	C15-C17,C21,C24, C26	Cancers	All other cancers
Malignant neoplasm of gallbladder	C23	Cancers	DM-related cancers
Malignant neoplasm of pancreas	C25	Cancers	DM-related cancers
Malignant neoplasms of lymphoid, haematopoietic and related tissue	C81-C96	Cancers	All other cancers
Malignant neoplasm of trachea, bronchus and lung	C33-C34	Cancers	All other cancers
Malignant neoplasm of prostate	C61	Cancers	All other cancers
Malignant neoplasm of breast	C50	Cancers	DM-related cancers
Malignant neoplasm of cervix	C53	Cancers	All other cancers
Malignant neoplasm of corpus uteri	C54	Cancers	DM-related cancers
All other Neoplasms	C00-C14, C27-C32, C35-C49, C51-C52, C55-C60, C62-C80, C97-D48	Cancers	All other cancers
Respiratory	J00-J98, J19, J22-J99	Respiratory, Renal & Liver	Respiratory

Respiratory infection	J12-J18, J09-J12, J20-21, A15-A19	Infections	Respiratory infection
Diabetes	E10-E14, excluding E10.0, E10.1, E11.0, E11.1, E12.0, E12.1, E13.0, E13.1, E14.0, E14.1,	Diabetes	Diabetes
Acute Myocardial Infarction	I21-I22	Vascular	Acute Myocardial Infarction
Other Ischaemic Heart Disease & heart failure	I20, I23-I25, I50	Vascular	Ischaemic Heart Disease
Stroke (cerebrovascular)	I60-I69	Vascular	Stroke
Major Lower limb Amputations	OPCS codes: 093-095, 098-099	Amputations	Major Amputations
Minor Lower limb Amputations	OPCS codes: 101, 104, 108-109, 111-112, 118-119	Amputations	Minor Amputations
Hyperglycaemic crisis	E10.0, E10.1, E11.0, E11.1, E12.0, E12.1, E13.0, E13.1, E14.0, E14.1,	Diabetes	Hyperglycaemic crisis
Renal disease	N00-N09, N12-N14, N17, N19-N28	Respiratory, Renal & Liver	Renal disease
ESRD	N18	Respiratory, Renal & Liver	Renal disease
Kidney infection	N10-11, N15-N16, N30, N39	Infections	Kidney infection
Liver disease	K70-77	Respiratory, Renal & Liver	Liver disease
Skin, soft tissue and bone infections	L03, L97, I96, M86, B35-49	Amputations	Skin and bone infections
Sepsis	A40-A41	Sepsis	Sepsis

Supplemental Table 2. Proportional contribution to hospitalisation burden of leading cause-specific diseases in those with diabetes from 2003 to 2018.

**Men**	**2003**	**2004**	**2005**	**2006**	**2007**	**2008**	**2009**	**2010**
IHD	20.2%	19.4%	18.5%	17.6%	17.1%	16.5%	15.9%	15.3%
Stroke	4.9%	4.9%	4.9%	4.8%	4.7%	4.6%	4.5%	4.3%
AMI	5.8%	5.4%	5.1%	4.8%	4.6%	4.4%	4.2%	4.0%
All other cancers	14.4%	14.9%	15.3%	15.7%	16.0%	16.3%	16.5%	16.7%
DM-related cancers	2.3%	2.5%	2.6%	2.8%	2.8%	2.8%	2.8%	2.8%
Diabetes	13.8%	13.0%	12.2%	11.4%	11.0%	10.7%	10.3%	9.9%
Hyperglycaemic crisis	4.6%	4.8%	5.0%	5.3%	5.5%	5.7%	5.9%	6.2%
Major Amputations	1.5%	1.5%	1.4%	1.3%	1.2%	1.0%	0.9%	0.8%
Minor Amputations	1.9%	1.9%	1.8%	1.7%	1.6%	1.6%	1.5%	1.4%
Respiratory infections	4.3%	4.6%	4.9%	5.3%	5.6%	6.0%	6.3%	6.7%
Kidney infections	3.8%	4.2%	4.7%	5.3%	5.5%	5.8%	6.0%	6.3%
Skin, bone infections	4.4%	4.5%	4.6%	4.6%	4.6%	4.6%	4.7%	4.7%
Sepsis	0.6%	0.7%	0.8%	1.0%	1.1%	1.3%	1.5%	1.7%
Respiratory disease	10.4%	10.5%	10.7%	10.8%	10.8%	10.8%	10.8%	10.8%
Renal disease	4.0%	4.2%	4.4%	4.6%	4.7%	4.9%	5.0%	5.2%
CKD	2.0%	2.0%	2.0%	1.9%	1.9%	1.9%	1.8%	1.8%
Liver disease	1.1%	1.1%	1.1%	1.1%	1.2%	1.2%	1.3%	1.4%
**Women**	**2003**	**2004**	**2005**	**2006**	**2007**	**2008**	**2009**	**2010**
IHD	16.8%	16.1%	15.3%	14.6%	13.9%	13.3%	12.6%	12.0%
Stroke	5.7%	5.6%	5.6%	5.5%	5.4%	5.3%	5.2%	5.0%
AMI	5.0%	4.8%	4.5%	4.2%	4.0%	3.9%	3.7%	3.5%
All other cancers	11.1%	11.4%	11.7%	11.9%	12.1%	12.3%	12.5%	12.6%

DM-related cancers	4.4%	4.7%	4.9%	5.1%	5.1%	5.0%	5.0%	4.9%
Diabetes	11.5%	10.8%	10.0%	9.3%	8.9%	8.4%	8.0%	7.6%
Hyperglycaemic crisis	7.4%	7.6%	7.7%	7.9%	8.1%	8.3%	8.5%	8.7%
Major Amputations	1.0%	0.8%	0.7%	0.6%	0.6%	0.5%	0.5%	0.5%
Minor Amputations	1.1%	1.0%	0.9%	0.8%	0.7%	0.7%	0.7%	0.7%
Respiratory infections	4.6%	4.9%	5.3%	5.7%	6.1%	6.5%	6.9%	7.3%
Kidney infections	8.7%	9.2%	9.8%	10.3%	10.7%	11.2%	11.6%	12.0%
Skin, bone infections	4.8%	4.8%	4.8%	4.7%	4.7%	4.6%	4.5%	4.5%
Sepsis	0.8%	0.9%	1.0%	1.2%	1.4%	1.6%	1.8%	2.0%
Respiratory disease	12.0%	12.2%	12.4%	12.5%	12.6%	12.7%	12.7%	12.8%
Renal disease	3.1%	3.2%	3.4%	3.5%	3.6%	3.7%	3.9%	4.0%
CKD	1.4%	1.3%	1.3%	1.3%	1.2%	1.2%	1.1%	1.1%
Liver disease	0.7%	0.8%	0.8%	0.9%	0.9%	0.9%	0.9%	0.9%
**Men**	**2011**	**2012**	**2013**	**2014**	**2015**	**2016**	**2017**	**2018**
IHD	15.1%	14.8%	14.5%	14.2%	13.8%	13.4%	13.0%	12.6%
Stroke	4.3%	4.2%	4.2%	4.1%	4.0%	3.8%	3.6%	3.5%
AMI	4.1%	4.2%	4.2%	4.3%	4.2%	4.1%	4.0%	3.9%
All other cancers	17.1%	17.4%	17.7%	18.0%	18.2%	18.4%	18.6%	18.7%
DM-related cancers	2.7%	2.6%	2.5%	2.4%	2.4%	2.4%	2.4%	2.4%
Diabetes	8.7%	7.7%	6.7%	5.9%	5.8%	5.7%	5.6%	5.5%
Hyperglycaemic crisis	6.0%	5.8%	5.6%	5.5%	5.4%	5.3%	5.3%	5.2%
Major Amputations	0.8%	0.7%	0.6%	0.6%	0.6%	0.6%	0.5%	0.5%
Minor Amputations	1.4%	1.4%	1.4%	1.3%	1.3%	1.3%	1.3%	1.2%

Respiratory infections	7.2%	7.6%	8.1%	8.5%	9.0%	9.5%	10.0%	10.6%
Kidney infections	6.6%	6.9%	7.1%	7.4%	6.9%	6.4%	5.9%	5.5%
Skin, bone infections	4.7%	4.7%	4.7%	4.8%	4.8%	4.7%	4.7%	4.7%
Sepsis	2.0%	2.4%	2.8%	3.2%	3.7%	4.3%	5.0%	5.8%
Respiratory disease	10.8%	10.9%	10.9%	10.9%	10.8%	10.8%	10.7%	10.6%
Renal disease	5.4%	5.5%	5.7%	5.9%	6.0%	6.2%	6.3%	6.5%
CKD	1.8%	1.7%	1.7%	1.7%	1.6%	1.6%	1.6%	1.5%
Liver disease	1.4%	1.4%	1.4%	1.4%	1.4%	1.4%	1.4%	1.4%
**Women**	**2011**	**2012**	**2013**	**2014**	**2015**	**2016**	**2017**	**2018**
IHD	11.5%	11.0%	10.5%	10.0%	9.8%	9.6%	9.4%	9.2%
Stroke	5.0%	4.9%	4.8%	4.7%	4.5%	4.3%	4.1%	3.9%
AMI	3.4%	3.4%	3.3%	3.2%	3.1%	3.0%	2.9%	2.7%
All other cancers	13.0%	13.3%	13.6%	13.9%	14.2%	14.4%	14.6%	14.8%
DM-related cancers	4.9%	4.9%	4.9%	4.9%	4.9%	4.9%	4.8%	4.8%
Diabetes	6.7%	5.9%	5.2%	4.5%	4.3%	4.1%	3.9%	3.7%
Hyperglycaemic crisis	8.5%	8.3%	8.1%	7.9%	7.7%	7.6%	7.4%	7.2%
Major Amputations	0.5%	0.4%	0.4%	0.3%	0.3%	0.3%	0.2%	0.2%
Minor Amputations	0.6%	0.6%	0.5%	0.5%	0.5%	0.5%	0.5%	0.5%
Respiratory infections	7.8%	8.4%	9.1%	9.7%	10.4%	11.0%	11.7%	12.4%
Kidney infections	12.1%	12.1%	12.1%	12.1%	11.3%	10.5%	9.7%	9.0%
Skin, bone infections	4.5%	4.5%	4.4%	4.4%	4.4%	4.3%	4.2%	4.2%
Sepsis	2.3%	2.7%	3.1%	3.5%	4.0%	4.6%	5.3%	6.0%
Respiratory disease	13.0%	13.2%	13.4%	13.6%	13.7%	13.8%	13.9%	13.9%

Renal disease	4.2%	4.3%	4.5%	4.7%	4.9%	5.0%	5.2%	5.3%
CKD	1.1%	1.0%	1.0%	1.0%	0.9%	0.9%	0.9%	0.8%
Liver disease	1.0%	1.0%	1.1%	1.1%	1.2%	1.2%	1.2%	1.2%

Supplemental Figure 1. Example annual exposure periods for patient having hospital admission for AMI, Cancer, Respiratory infection respectively.

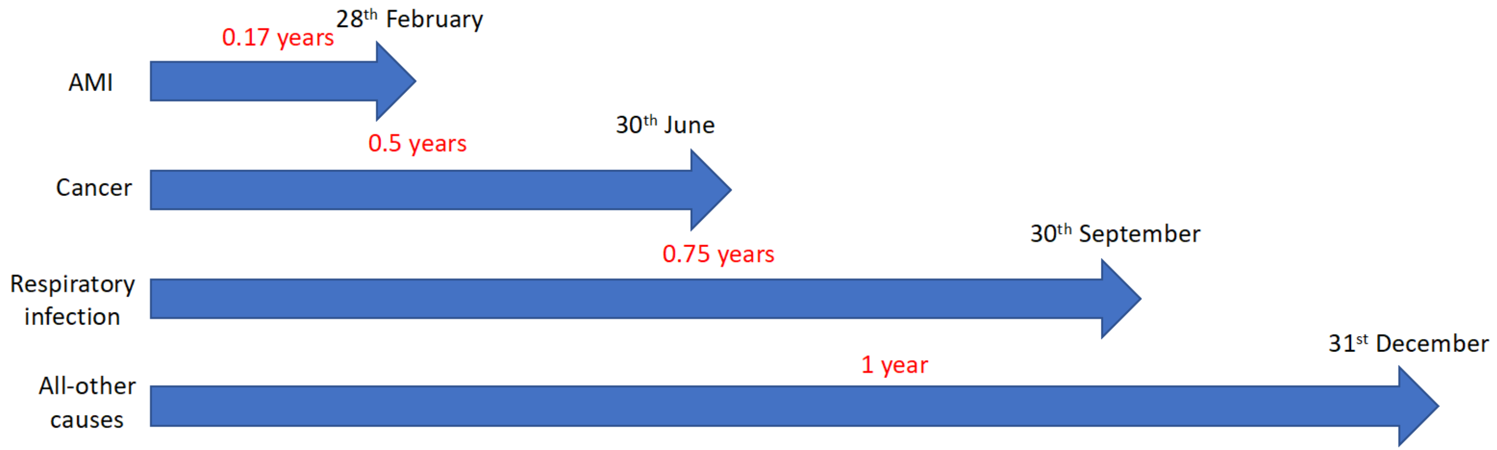

Supplemental Figure 2. Absolute change in cause-specific hospitalisation rates from 2003 to 2018 among those with diabetes stratified by age group.

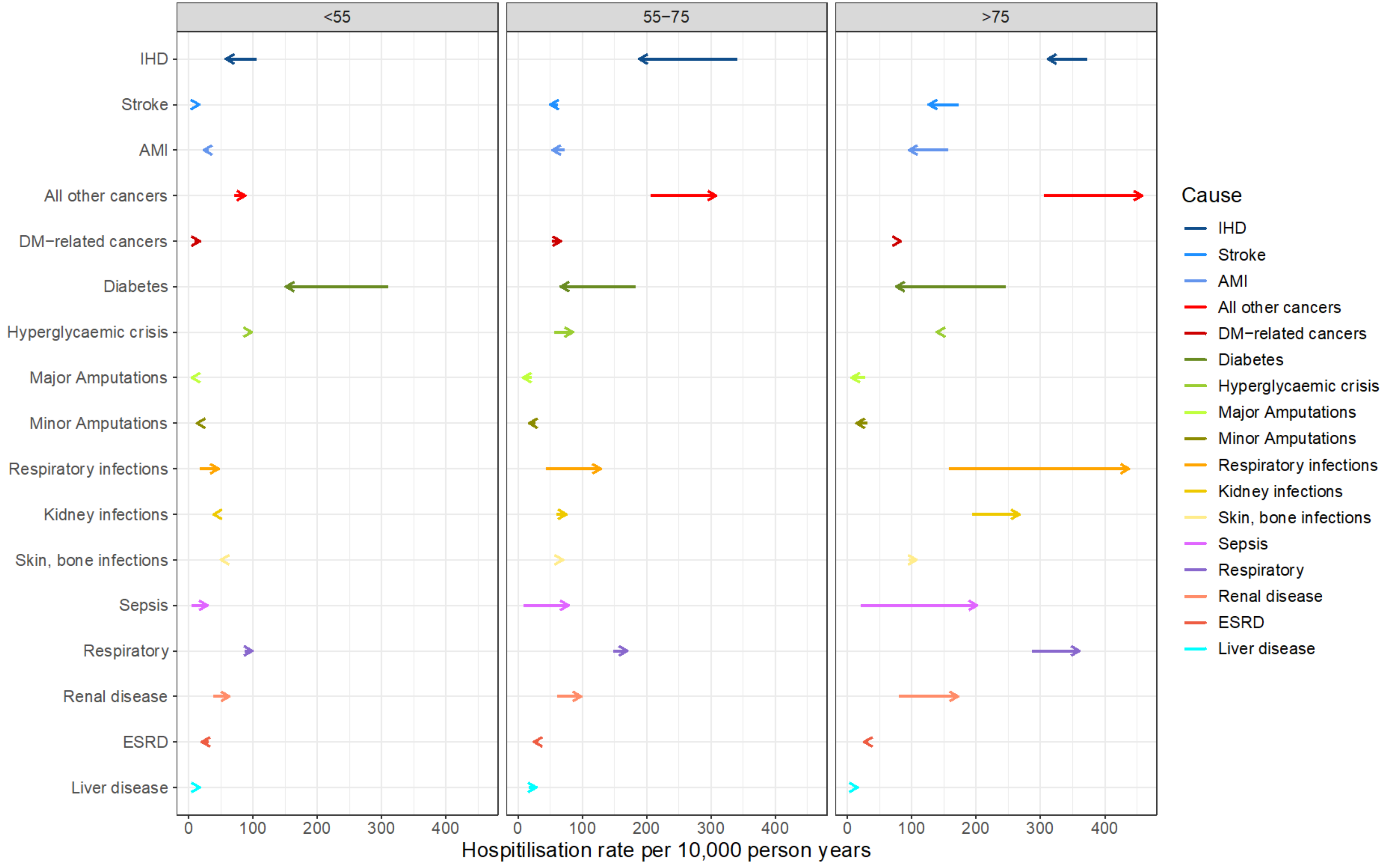

Supplement: Supplementary appendix [file mmc1.pdf]
